# Evolutionary methods for variable selection in the epidemiological modeling of cardiovascular diseases

**DOI:** 10.1186/s13040-018-0180-x

**Published:** 2018-08-14

**Authors:** Christina Brester, Jussi Kauhanen, Tomi-Pekka Tuomainen, Sari Voutilainen, Mauno Rönkkö, Kimmo Ronkainen, Eugene Semenkin, Mikko Kolehmainen

**Affiliations:** 1 Department of Environmental and Biological Sciences, University of Eastern Finland, Yliopistonranta 1 E, 70211 Kuopio, Finland; 2 Institute of Computer Science and Telecommunications, Reshetnev Siberian State University of Science and Technology, Krasnoyarsky Rabochy ave. 31, Krasnoyarsk, 660037 Russia; 3 Institute of Public Health and Clinical Nutrition, University of Eastern Finland, Yliopistonranta 1 C, 70211 Kuopio, Finland

**Keywords:** Variable selection, Cardiovascular disease, Predictive modeling, Kuopio ischemic heart disease risk factor study

## Abstract

**Background:**

The redundancy of information is becoming a critical issue for epidemiologists. High-dimensional datasets require new effective variable selection methods to be developed. This study implements an advanced evolutionary variable selection method which is applied for cardiovascular predictive modeling. The epidemiological follow-up study KIHD (Kuopio Ischemic Heart Disease Risk Factor Study) was used to compare the designed variable selection method based on an evolutionary search with conventional stepwise selection. The sample contains in total 433 predictor variables and a response variable indicating incidents of cardiovascular diseases for 1465 study subjects.

**Results:**

The effectiveness of variable selection methods was investigated in combination with two models: Generalized Linear Logistic Regression and Support Vector Machine. We managed to decrease the number of variables from 433 to 38 and save the predictive ability of the models used. Their performance was evaluated with an F-score metric. At most, we gained 65.6% and 67.4% of the F-score before and after variable selection respectively. All the results were averaged over 5-folds of a cross-validation procedure.

**Conclusions:**

The presented evolutionary variable selection method allows a reduced set of variables to be chosen which are relevant to predicting cardiovascular diseases. A reference list of the most meaningful variables is introduced to be used as a basis for new epidemiological studies. In general, the multicollinearity of variables enables different combinations of predictors to be used and the same performance of models to be attained.

**Electronic supplementary material:**

The online version of this article (10.1186/s13040-018-0180-x) contains supplementary material, which is available to authorized users.

## Background

Epidemiological research aims to construct better understanding of the complexities in health and disease etiology. Nowadays, this means increasingly large amounts of data, especially in the predictive modeling of health risks and possible outcomes [1]. One of the obvious ways to tackle this *Big Data* problem is based on the involvement of powerful machines with high computational capacity. In theory, the technology would allow a tremendously big number of *variables* (other terms such as *features, predictors* or *attributes* are also used elsewhere) to be engaged in the datasets under study. The model performance, however, may not be improved by adding more and more variables. One reason for this is the redundancy of information [2, 3]. With respect to the translational aspects of epidemiological research, such as developing evidence-based diagnostic tools, it is impractical to consider study designs that require a multitude of input variables and clinical tests to gather a high-dimensional vector of variables for all of the patients to be checked. In this paper, we argue that epidemiologists have encountered a complex variable selection problem, which cannot be completely solved with traditional computational methods, and we suggest an alternative solution to this problem.

Conventional approaches for variable selection in epidemiological modeling include two general classes of methods: prior knowledge-based and automated [4]. Prior knowledge-based methods use a priori information about the variable relevance from previous literature and utilize the results of earlier studies. However, it is not clear to what extent we can apply the results of other particular cases to our purposes. Have the other study samples been representative enough, or for that matter, how specific is our own study?

One may decide to involve experts to choose variables, but in the case of complicated high dimensional datasets, this human-operated approach becomes very difficult or impossible to apply.

The most frequently used methods, referred to as computer-driven or the automated selection of variables to be used in the modeling, are backward elimination, forward selection and stepwise selection of variables [5]. All these procedures work iteratively adding or removing one variable at a time until the pre-specified stopping rule is satisfied. They do not estimate the accumulative contribution of several variables to the model. Furthermore, it has been shown that these methods are sensitive to random fluctuations in the dataset when using bootstrap samples [6]. This implies that the particular predictors selected with these automated methods for a given database might be inappropriate for others.

In recent times, some research groups have become aware of these challenges in epidemiology. It has been shown, for example, that traditional approaches have not performed well in the experiments with large-scale datasets [7]. As a result, there have been a number of studies trying to apply some alternative methods such as shrinkage or penalized regression [8]. These techniques were proposed two decades ago but, according to Walter and Tiemeier’s study, in 2008 there were no publications in epidemiological journals using these methods [4]. Nevertheless, in some recent reports it has been claimed that the Least Absolute Shrinkage and Selection Operator (LASSO) is applicable and effective for high-dimensional datasets [9, 10]. Even though some promising results have been obtained, LASSO logistic regression is applied quite rarely in current epidemiological studies [11]. Besides, some researchers highlight its bias towards false positives [12]. All these examples distinctly underpin the necessity to develop novel variable selection methods able to cope with large-scale data. In [3] the authors have made an extensive survey on how to apply data-mining methods in epidemiological studies and their reasoning points to the same directions as ours. However, their results are lacking the evolutionary approach which we have found important in this study in finding the most effective combinations of multicollinear variables.

In this article, we introduce an advanced variable selection method which is based on an evolutionary search. We apply a genetic algorithm (GA) to explore a high-dimensional variable space in an effective way. A linear increase of the number of variables leads to an exponential growth of possible variable combinations. However, compared to many algorithms and methods, GAs are robust to ‘the curse of dimensionality’ and, therefore, might be successfully used to select relevant variables [13].

We have investigated the performance of the proposed method on a population-based epidemiological KIHD (Kuopio Ischemic Heart Disease Risk Factor Study) dataset, containing the state vectors of 433 characteristics regarding the study subjects (*N* = 1465). Firstly, we have managed to reduce the dimensionality of the input vector from 433 to 38 variables without damage to the performance of predictive models. Then, we have created a ranking system for all of the variables based on their relevance to cardiovascular diseases (CVDs). To be more precise, the aim of this article is to introduce the evolutionary variable selection method and discuss the most relevant selected variables. Finally, we have revealed that due to the multicollinearity of variables, the same performance of models might be achieved with many different combinations of predictors. We propose that this may carry significant implications for both the theoretical and clinical use of epidemiological data.

## Methods

### Evaluated models

In this research, we investigate variable selection methods in combination with two models: Generalized Linear Logistic Regression and Support Vector Machine.

*Logistic Regression (Logit)* is a type of linear model which is used to describe the relationship between a binary response (dependent) variable *Y* and several predictor (independent) variables *X*_*1*_*, X*_*2*_*, ..., X*_*n*_ [14]. Essentially, a logistic regression expresses the conditional probability *P*(*Y*  =  *1* |  **X**  =  **x**) on the assumption that an outcome variable is a stochastic event: in diagnostics, *Y* = *1* usually means the presence of a disease. Formally, it is defined as follows:

1 $$ \mathit{\log}\left(\frac{P\left(Y= 1|\kern0.5em \mathbf{X}=\mathbf{x}\right)}{1-P\left(Y= 1|\mathbf{X}=\mathbf{x}\right)}\right)={\upbeta}_0+{\upbeta}_1{x}_1+\dots +{\upbeta}_n{x}_n; $$

2 $$ P\left(Y= 1|\mathbf{X}=\mathbf{x}\right)=\frac{1}{{}_{1\kern0.5em +\kern0.5em e}-\left({\upbeta}_0+{\upbeta}_1{x}_1+\dots +{\upbeta}_n{x}_n\right)}. $$

To evaluate coefficients *β*_*i*_ the maximum likelihood estimate is used: the parameters should maximize the probability of the observed cases. The fitted model (2) allows predictions to be obtained based on the decision rule: *Y* = *1* if *P(Y* = *1*|**X** = **x***)* ≥ *0.5* and *Y* = *0* if *P(Y = 1|***X** = **x***)<0.5*. A default cutoff value 0.5 might be varied to achieve a better result for each particular problem.

Results obtained with a logistic regression model are easily interpreted. However, the use of this model is not recommended when independent variables are highly correlated and their number is quite large: in this case, parameter estimators become unstable [15].

*Support Vector Machine (SVM)* is a more complex model which is based on designing hyperplanes **w** ⋅ **x** + *b* = *0* that work as decision boundaries [16]. The main concept of the SVM algorithm is to construct the optimal hyperplane that maximizes the margin between two different groups of objects (in our study an object means a patient or subject). The term *margin* correspondingly means the distance to the closest training point.

The essential advantage of this model is the ability to cope with a non-linearly separable dataset with the usage of loss and kernel functions. Loss functions penalize misclassified cases, whereas kernel functions map data into a higher dimensional space where linear separation is possible.

Generally, training SVM models is accomplished through minimizing the error function:

3 $$ \frac{1}{2}{\mathbf{w}}^T\cdot \mathbf{w}+C\cdot \underset{i\kern0.5em =\kern0.5em 1}{\overset{N}{\Sigma}}{\xi}_i\to \mathit{\min}, $$

which is subject to the constraints:

4 $$ {y}_i\left({\mathbf{w}}^T\upphi \left({\mathbf{x}}_i\right)+b\right)\ge 1-{\xi}_i\kern0.5em \mathrm{and}\kern0.5em {\xi}_i\ge 0,i= 1,\dots, N, $$

where *C* is an adjustable parameter, *ξ*_*i*_ expresses an error *max*(*0*, *1* ‐ *y*_*i*_ ⋅ (**w**^*T*^**x**_*i*_ + *b*)) on training examples **x**_*i*_, *y*_*i*_ where *y*_*i*_ ∈  ± 1, and ϕ(…) is a kernel function. In our study, we use polynomial kernels and, to design a hyperplane separating sets of examples, Sequential Minimal Optimization (SMO) is applied for solving the large-scale quadratic programming problem [17].

As an alternative variable selection method, we investigated a traditional *stepwise selection* [18] to demonstrate the advances of our approach. Stepwise selection works as a combination of backward elimination and forward selection. Starting with an empty set of predictors, it adds to the model one variable at a time (as in forward selection). However, at each iteration an included variable might also be removed from the model if it is not significant any more. A pre-specified criterion is used to stop the variable selection process.

Stepwise selection is rather economical in the sense of computational costs but this iterative strategy is likely to miss the optimal model. Moreover, as a result of deleting insignificant predictors, the significance of the remaining variables is revalued and often becomes exaggerated, which is misleading [5].

In our modeling, we also assign ranks to all variables based on the order in which they were selected. Thus, assuming that *N* variables are selected, the first feature gets the highest score equal to *N*, whereas the last variable gets the lowest score which is equal to 1. Variables that are not selected receive a 0 score. If a variable is removed at any iteration of stepwise selection, it also receives a 0 score. These ‘raw’ scores are transformed to the interval [0,1] by using a linear normalization.

### Evolutionary variable selection

We designed our variable selection method on the basis of a filter approach. As opposed to wrapper or embedded techniques, this method is beneficial for large-scale datasets because it does not involve any model to evaluate combinations of variables [19] and, therefore, requires fewer computational resources. In essence, filtering precedes modeling and corresponds to the preprocessing stage.

There are some statistical criteria which might be optimized in the framework of the filter approach: Attribute Class Correlation (AC), Inter- and Intra- Class Distances (IE and IA), Laplasian Score (LS), and Representation Entropy (RE) [20]. Previously, we tested various combinations of these criteria and found the most appropriate two-objective model [21]:

5 $$ IA\kern0.5em =\kern0.5em \frac{1}{n}\underset{r\kern0.5em =\kern0.5em 1}{\overset{k}{\Sigma}}\underset{j\kern0.5em =\kern0.5em 1}{\overset{n_r}{\Sigma}}d\left({p}_j^r,\kern0.5em {p}_r\right)\kern0.5em \to \min, $$

6 $$ IE\kern0.5em =\kern0.5em \frac{1}{n}\underset{r\kern0.5em =\kern0.5em 1}{\overset{k}{\Sigma}}{n}_rd\left({p}_r,\kern0.5em p\right)\kern0.5em \to \min, $$

where ${p}_j^r$ is the *j*^*th*^ example from the *r*^*th*^ class, *p* is the central example of the data set, *d*(...,...) denotes the Euclidian distance, *p*_*r*_ and *n*_*r*_ represent the central example and the number of examples in the *r*^*th*^ class. In our experiments, *p* and *p*_*r*_ are assigned as vectors of average values of variables calculated on the whole set and on the *r*^*th*^ class, respectively. Criteria 5 and 6 are optimized at once, using a Pareto-dominance idea. We do not apply any convolution of criteria to reduce this two-objective problem to a single-objective one. Therefore, there is no need to assign weights of criteria.

To define reduced vectors of variables satisfying the chosen criteria in an optimal way, we suggest applying a multi-objective genetic algorithm (MOGA). Generally, GAs are universal, flexible, and widely used [22]. Due to their unique abilities, GAs remain the only applicable tool for many complex optimization problems. They can be effectively employed for high-dimensional domains with different types of variables (continuous, discrete, or any other non-numerical variables presented as a binary string). It is even possible to use these algorithms in the dynamic environment when the optimum is changing. In short, GAs imitate the alternation of generations based on the principles of natural selection: a fitness function reflects optimization criteria, and genetic operators are applied to produce offspring (new candidate solutions) and direct a search towards prospective regions.

**Fig. 1:**
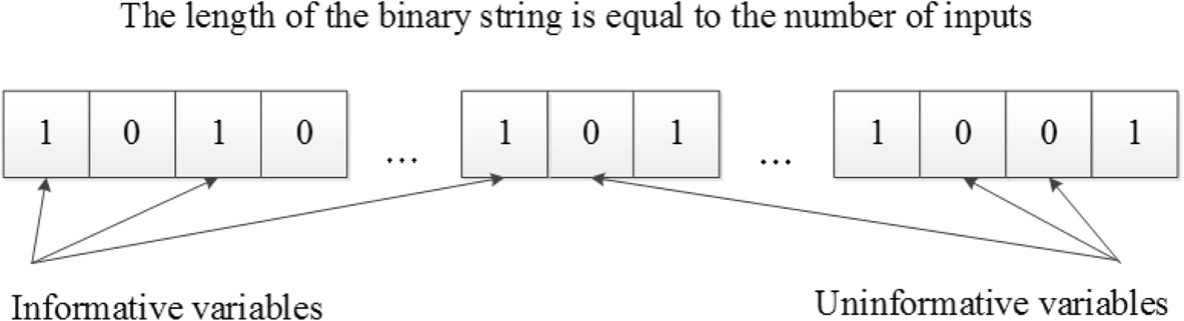
The binary representation of a reduced variable set. One corresponds to a variable that is present in the model input and zero corresponds to an ignored variable

In our approach, the population of candidate solutions contains reduced vectors of variables. Each binary string (a so-called chromosome) codes a combination of selected variables in the following way: *one* and *zero* correspond to a relevant variable and an irrelevant one respectively (Fig. 1). At every generation, all individuals from the population are assessed with the criteria (5) and (6). During the algorithm execution, possible combinations of variables are evaluated together. In other words, the GA ignores the significance of an individual variable and this distinguishes it from conventional iterative methods.

The binary representation of a reduced variable set. One corresponds to a variable that is present in the model input and zero corresponds to an ignored variable.

To achieve a high level of effectiveness of an evolutionary search, we applied a modified cooperative MOGA including three different methods [23]: Non-dominated Sorting Genetic Algorithm II (NSGA-II) [24], Preference-Inspired Co-Evolutionary Algorithm with goal vectors (PICEA-g) [25], and Strength Pareto Evolutionary Algorithm 2 (SPEA2) [26]. These algorithms are based on different heuristic strategies, which allows us to preserve the diversity of candidate solutions. Moreover, they work in a parallel way, which saves computational time.

It is well known that an outcome of a MOGA is a set of non-dominated points which form a Pareto set approximation: for our problem, it is a set of alternative variable combinations. To derive the final solution we took into account all non-comparable variable vectors from the Pareto set. However, GAs apply heuristics and may lead to different (appropriate but not always optimal) solutions in each run. Therefore, we decided to launch the cooperative MOGA several times (specifically, 15) on the training set of every fold to get ‘representative’ variables. In each run, the final Pareto set approximation contained 30 candidate solutions. Thus, we collected 30∙15 = 450 binary strings coding reduced variable sets. Then, for each variable we estimated the relative number of cases when it was chosen and based on these scores we assigned ranks for each variable. The final reduced vector of variables comprised of variables with absolute ranks (i.e. 1). Additionally, we compared the model performance on a number of separate solutions obtained by the MOGA in different runs with its performance on the set of variables having absolute ranks: the results were similar.

### Database description

The epidemiological follow-up study, KIHD, was launched in 1984 and is still continuing. It comprises of a population sample of 2682 middle-aged men recruited in 1984–1989, and 920 ageing women recruited in 1998–2001 from the city of Kuopio and its surrounding communities in Eastern Finland [27–29]. The sample is one of the most thoroughly characterized epidemiological study populations in the world, with thousands of biomedical, psychosocial, behavioral, clinical and other variables. Over the past 30 years, the KIHD study has proven to be a valuable source for epidemiological research, and it has yielded over 500 original peer reviewed articles in international scientific journals. Follow-up CVD diagnoses were collected with record linkage to the national computerized Hospital Discharge Register and to the national computerized Causes of Death Register. The focus in the KIHD study originally was on CVDs, and especially on ischemic heart disease, but also a wide range of other health outcomes have been examined.

A subset of 433 predictor variables was preselected from the baseline data (1984–1989, it consists of only male study subjects) to represent different types of variables: anthropometric, biochemical, behavioral and nutritional. In this research, we consider only CVDs as a response variable. The dataset was preprocessed in the following way:

- Firstly, study subjects who had a history of any CVD problems (before or at the baseline time point) were excluded so that we got vectors of variables for only those people who were free of obvious diseases at the beginning of the follow-up period;
- Secondly, subjects who had been free of disease at the baseline but then died due to any non-CVD reason, were also excluded.

After these two steps, we obtained a dataset with 1465 study subjects (602 sick and 863 healthy subjects). The final step of preprocessing resulted in two main groups of subjects: people who had any new serious incident of CVDs during the follow-up until 2012–2013 were categorized as ‘unhealthy’ (i.e. the response variable is equal to 1), and those who did not face CVDs during the same period were categorized as ‘healthy’ (i.e. the response variable is equal to 0).

Hence, the general purpose of predictive modeling aims at distinguishing between these two groups (‘healthy’ and ‘unhealthy’).

## Results

To investigate the effectiveness of the considered variable selection methods and to estimate the performance of predictive models, we implemented a 5-fold cross-validation procedure with stratification so that for each out of 5 runs we had training and test samples. The results of predictive modeling were processed to get confusion matrixes and, finally, we evaluated an F-score metric: 0% corresponds to the worst performance, whereas 100% implies the best quality of prediction [30].

In the beginning, we applied a Linear Logistic Regression and SVM models for the set of all 433 variables [31]. The main purpose of this experiment was to determine whether non-linear models were more beneficial for the KIHD data on the full set of variables. For the Logistic Regression model, we tested different cutoffs and found that changing a default value 0.5 did not provide us with the better result. We trained three SVM models, the degree of the polynomial kernel was equal to 1.0 (linear one), 1.5 and 2.0 (non-linear ones). We also tested SVM models with other degrees of the polynomial kernel, but we obtained approximately the same or even worse result.

**Fig. 2:**
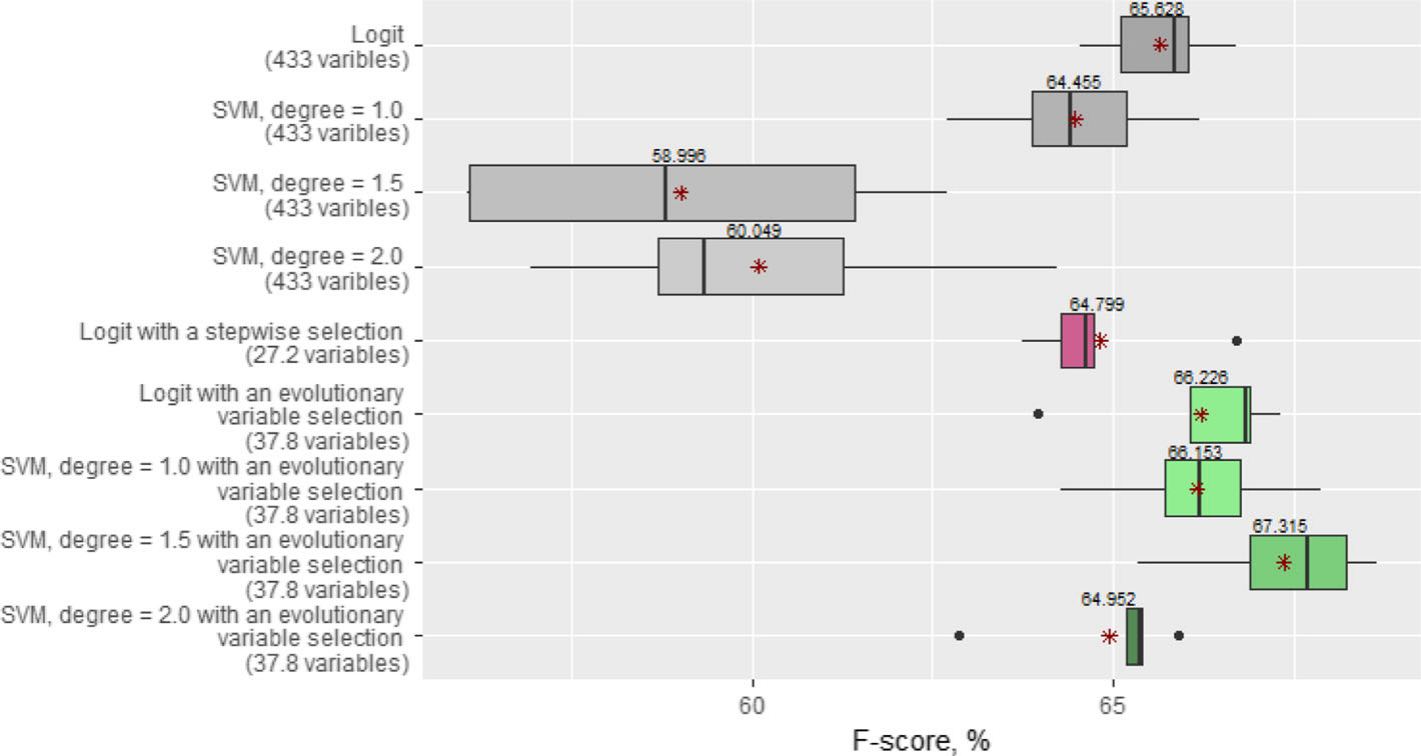
The performance of cardiovascular predictive modeling in combination with variable selections. The figure shows boxplots that compare F-score values obtained with Logit and SVM (the degree of the polynomial kernel is 1.0, 1.5 and 2.0) models without any variable selection, with stepwise selection and with evolutionary variable selection. Mean F-score values are marked with asterisks

Based on the F-score values we discovered that for the current dataset the usage of more complex SVM models (SVM, degree = 1.5 or 2.0) did not lead to better results: the highest F-score value averaged over 5 folds was gained with linear models (SVM, degree = 1.0 and Logit) and was about 64.5–65.6% (Fig. 2). The distribution of subjects in the confusion matrixes obtained with these linear models was slightly different (confusion matrixes are available in Additional file 1: Tables).

The performance of cardiovascular predictive modeling in combination with variable selections. The figure shows boxplots that compare F-score values obtained with Logit and SVM (the degree of the polynomial kernel is 1.0, 1.5 and 2.0) models without any variable selection, with stepwise selection and with evolutionary variable selection. Mean F-score values are marked with asterisks.

Next, we used a conventional stepwise selection method to find a set of relevant variables. For each run, we received a reduced vector of variables that were used as inputs by the Logistic Regression. On average, the number of variables was reduced to 27.2 and on this set of selected variables, we could achieve 64.8% of the F-score metric with the Logistic Regression (Fig. 2). A table containing all of the variables with non-zero ranks is available in Additional file 2: Table S6.

Then we applied the proposed evolutionary variable selection method. As a result, we obtained a set with 37.8 relevant variables on average. The proposed method was developed in the framework of a filter approach so that we could use it in combination with different models. In our experiments, we tested the predictive ability of the Logistic Regression and SVM models on the obtained variable set (Fig. 2). For the Logistic Regression, the average F-score value increased slightly to 66.2%. With SVM models, we could achieve 67.3% of the F-score metric on average. We should note that on the reduced dataset, the highest F-score was achieved with the non-linear SVM (degree = 1.5).

**Fig. 3:**
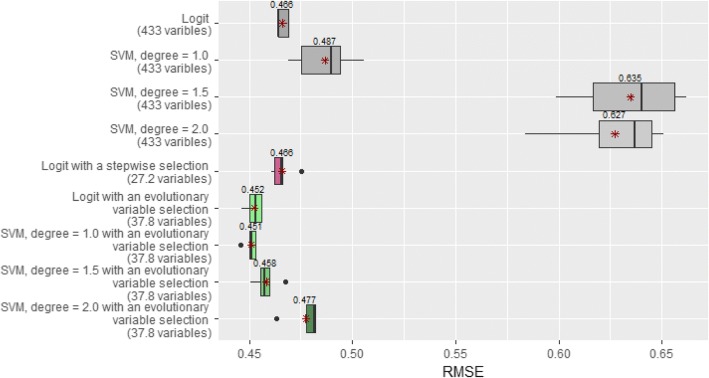
The performance of cardiovascular predictive modeling in combination with variable selections. The figure portrays boxplots that reflect RMSE (root mean square error) values obtained with Logit and SVM (the degree of the polynomial kernel is 1.0, 1.5 and 2.0) models without any variable selection, with stepwise selection and with evolutionary variable selection. Mean RMSE values are marked with asterisks

To perform a deeper analysis, in addition to model predictions (‘healthy’ or ‘unhealthy’), we registered the probabilities of CVDs and calculated a root mean square error (RMSE) (Fig. 3). For test instances with CVDs, actual probabilities were equal to 1 and for healthy subjects they were equal to 0. In comparison with the F-score, which operates only with the predictions ‘healthy’ and ‘unhealthy’, this metric allows us to take into account the difference between an estimated probability and its actual value.

The performance of cardiovascular predictive modeling in combination with variable selections. The figure portrays boxplots that reflect RMSE (root mean square error) values obtained with Logit and SVM (the degree of the polynomial kernel is 1.0, 1.5 and 2.0) models without any variable selection, with stepwise selection and with evolutionary variable selection. Mean RMSE values are marked with asterisks.

**Fig. 4:**
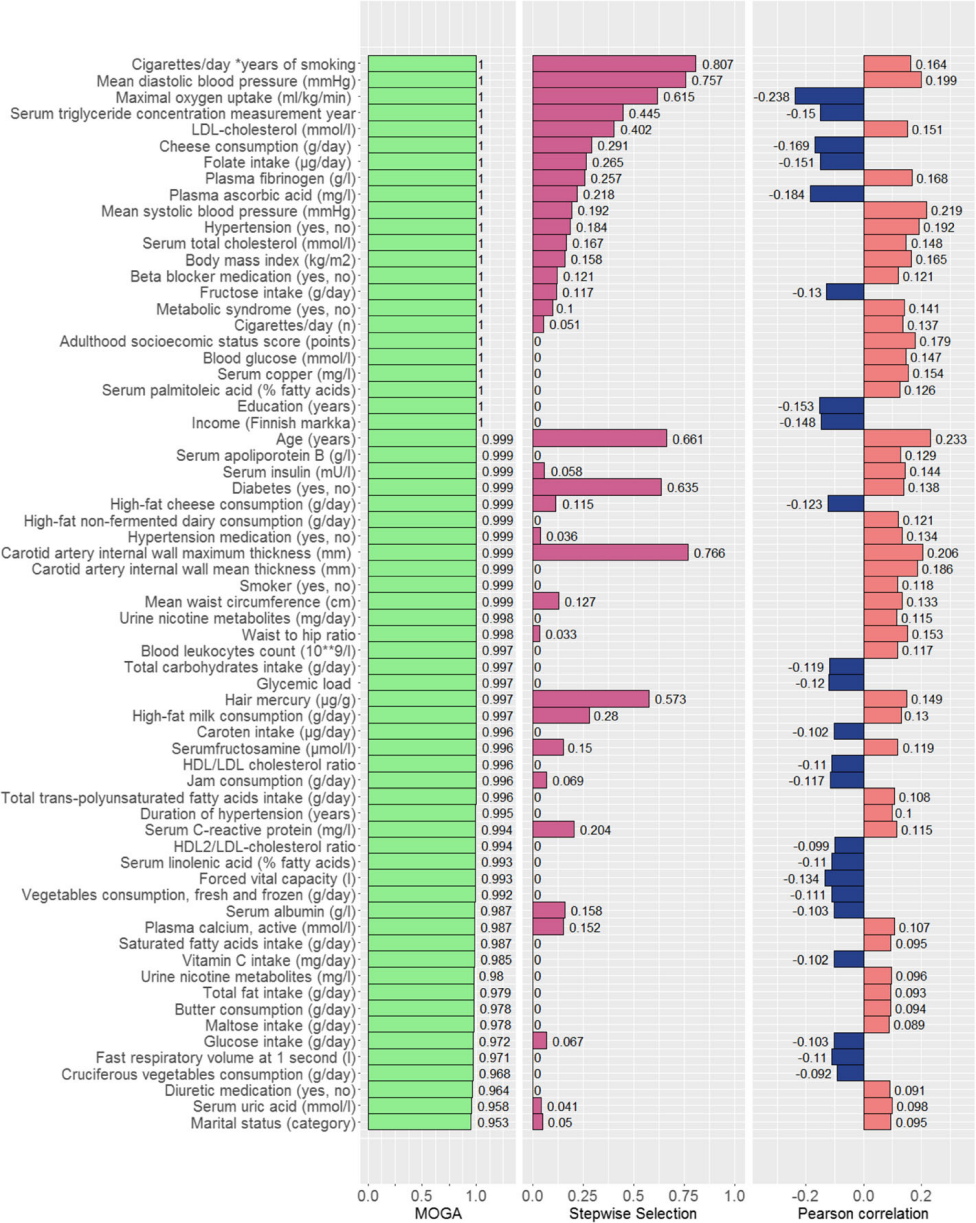
The list of variables whose MOGA-ranks are higher than 0.95. The figure shows ranks of the listed variables given by the MOGA, stepwise selection and Pearson correlation coefficients

As is described in the Methods section, after applying stepwise selection we also obtain ranks expressing the relevance of each variable in the dataset. In our experiment, the final ranks were averaged over 5 runs (Fig. 4, the central plot). Additionally, we computed Pearson correlation coefficients to show the basic association between the response and predictor variables (Fig. 4, the right plot).

Furthermore, we composed an alternative ranking system based on scores evaluated after the use of the MOGA (MOGA-ranks). In this system, many variables have a rather high rank because the MOGA determines a set of alternative solutions with different combinations of relevant variables. Figure 4 contains variables with MOGA-ranks ≥ 0.95. The extended list of variables with MOGA-ranks ≥ 0.9 is available in Additional file 3: Table S7.

The list of variables whose MOGA-ranks are higher than 0.95. The figure shows ranks of the listed variables given by the MOGA, stepwise selection and Pearson correlation coefficients.

In Fig. 4, we present all the ranks for ‘MOGA’, ‘Stepwise selection’, and ‘Pearson correlation’ so that it is possible to analyze if they agree with each other. Nevertheless, one should remember that a comparison of absolute values of different ranks is meaningless because they belong to various ranking systems.

## Discussion and conclusion

In this study, we have presented an advanced evolutionary variable selection method which has been applied to a high-dimensional epidemiological KIHD database. Although we have managed to reduce the number of variables significantly (from 433 to 38) without any damage to the predictive ability of the models used, the remaining concern is related to quite moderate F-score values. We used a traditional Logistic Regression, and linear (degree = 1.0) and non-linear (degree = 1.5; 2.0) SVM models. On the whole dataset (433 variables), Linear models (Logit and SVM, degree 1.0) provided us with approximately the same performance (F-score ≈ 65%), whereas non-linear SVM models demonstrated lower performance (F-score ≈ 59%). This shows that it is more difficult for a learning algorithm to adjust the parameters of more complex models having many input variables. Despite the similar values of the F-score metric for Logit and SVM (degree = 1.0), we may note that the use of these models leads to confusion matrixes which are different in the sense of false positive and false negative errors (see Additional file 1: Tables S3 and Table S5a). In terms of the RMSE metric, the linear models also outperformed the non-linear ones on the full dataset (Fig. 3).

The Logistic Regression with a conventional stepwise selection demonstrated an even slightly worse result (F-score ≈ 64.8%) than it showed with no variable selection. Conversely, after applying the evolutionary variable selection, all the considered models could achieve a higher F-score. Figure 3 also illustrates that in the sense of the RMSE metric the predictive ability of all the models used is significantly higher after the evolutionary variable selection. Moreover, on the reduced dataset we could gain the highest F-score ≈ 67.3% with the non-linear SVM model (degree = 1.5).

At the moment, distinguishing between ‘healthy’ and ‘unhealthy’ subjects has been performed at a general level by grouping different CVD diagnoses together. In future studies, various subtypes of CVD need to be studied separately. We suggest that designing ‘disease networks’ [32] may help us to reveal non-trivial connections among diverse CVD problems and, finally, to gain higher F-score values. Some advanced machine-learning techniques based on *Deep Learning* should be tested as they can successfully tackle high-dimensional problems [33], especially, if we want to test the several thousands of variables in the KIHD database together with detailed genetic information that is also available for part of the study cohort.

We have shown that the number of variables in the KIHD dataset might be reduced from hundreds to the order of tens of variables, which may have practical value. In prospect, the presented variable selection method should be examined on larger datasets.

In addition, our method is based on a filter approach, making it possible to combine it with two different models (a Logistic Regression and a SVM model) without re-executing all computations.

In general, it is accepted that in the development of CVDs and related adverse events, the subject characteristics such as age, gender, dyslipidemia, hypertension and obesity are important, as well as health behavioral characteristics such as smoking, physical activity and diet. When looking into the lists created by stepwise selection and MOGA (Additional file 2: Table S6 and Additional file 3: Table S7, respectively), many notions can be made. For instance, how the established CVD risk factors [34, 35] perform. In the stepwise selection Cigarettes/day*years of smoking, *Mean diastolic blood pressure*, *Age (years)*, and *LDL-cholesterol (mmol/l)* can be found among the first ten, in this order. In the MOGA, three of these can be found among those that have the score 1, besides age that received a score of 0.999. Other top-ten stepwise selection variables that received the score 1 in the MOGA were *Maximal oxygen uptake (ml/kg/min)*, which makes perfect sense, and *Serum triglyceride concentration measurement year*, which is a rather obscure variable to be that influential. Looking at the MOGA first, there are some other notions. Among those variables that have been selected by the model into each and every combination set (i.e. with the score 1), there are three socioeconomical status–related variables: *Income (Finnish markka), Adulthood socioecomic status score*, and *Education (years)*. This demonstrates very clearly the robustness of the model with regards to collinearity. Furthermore, all the three variables received a score of 0.000 in stepwise selection. Hence, we would like to highlight one of the main benefits of our proposal. Having hundreds or thousands variables which belong to different categories, it is not necessary to apply some preliminary analysis (like correlation-based or others), involve experts whose opinions are often biased, or investigate variables separately in each category. As a proper alternative, we offer just to unify all available variables and run the algorithm which is able to cope with variable selection effectively and quickly.

The confusion matrixes (Additional file 1: Table S1–Table S5) show that there are a fairly large number of subjects that the models were not able to classify correctly. This may represent a set of variables to choose from that is not optimal (i.e. important predictors missing from the dataset used), or a too heterogeneous, etiologically distinct outcome so that the models are actually trying to identify a set of variables that can classify several outcomes at the same time. However, there are some differences between the models that may be worth consideration. The MOGA-SVM model (Additional file 1: Table S5) gives the best specificity (with the degree of a polynomial kernel equal to 1.5) – the proportion of true negatives classified right –, even better than SVM with the full dataset (Additional file 1: Table S4).

It is necessary to emphasize that a MOGA finds a set of alternative variable combinations. Besides, by running the global heuristic search multiple times, we have collected many reduced vectors of variables. Thus, the ranking system designed based on these diverse solutions seems more thorough and advantageous, compared to the limited results of stepwise selection.

In this article, we introduce a reference list of the most meaningful variables of the KIHD study (Fig. 4) which might be used as a basis of new epidemiological research projects. This is a valuable contribution to the CVD predictive modeling research.

Furthermore, the obtained list of relevant variables is rather flexible. Owing to the multicollinearity of data, several variables contain similar information so that their different combinations may lead to the same performance of models. This fact gives an opportunity to choose ‘top’ variables which are economical or convenient to measure. This is important, when research has to be conducted with limited resources and funding constraints.

The presented evolutionary variable selection method and the achieved results may benefit clinical practice as well. Those health care systems which can operate diagnostic procedures with fewer inputs are not only cheaper but also faster and thus more cost-effective. They also provide more opportunities to support online diagnostics. Moreover, reducing the number of variables helps to simplify self-diagnostic tools and make them more easily available for the general public for independent health monitoring.

## Additional files

Additional file 1: **Table S1.** Confusion matrix for the logistic regression on the full dataset. **Table S2.** Confusion matrix for the logistic regression on the variables selected by the stepwise method. **Table S3.** Confusion matrix for the logistic regression on the features selected by the MOGA. **Table S4.** Confusion matrix for the SVM model on the full dataset. **Table S5.** Confusion matrix for the SVM model on the features selected by the MOGA. These files contain confusion matrixes of our experiments. (PDF 94 kb)

Additional file 2: **Table S6.** The list of selected variables with non-zero ranks based on stepwise selection. The file includes the list of variables and their MOGA and stepwise selection ranks with Pearson correlation values. (PDF 236 kb)

Additional file 3: **Table S7.** The list of variables with MOGA-ranks ≥ 0.9. (PDF 245 kb)
