# Supplementary material for: Evolutionary methods for variable selection in the epidemiological modeling of cardiovascular diseases
Source: BioData Min. 2018 Aug 14;11:18. doi: 10.1186/s13040-018-0180-x (PMC6092817; doi:10.1186/s13040-018-0180-x)
Supplement: Supplementary file 1 — Table S1. Confusion matrix for the logistic regression on the full dataset. Table S2. Confusion matrix for the logistic regression on the variables selected by the stepwise method. Table S3. Confusion matrix for the logistic regression on the features selected by the MOGA. Table S4. Confusion matrix for the SVM model on the full dataset. Table S5. Confusion matrix for the SVM model on the features selected by the MOGA. These files contain confusion matrixes of our experiments. (PDF 94 kb) [file 13040_2018_180_MOESM1_ESM.pdf]

Table 1. Confusion matrix for the logistic regression on the full dataset

	Sick_true	Healthy_true
Sick_predicted	321	198
Healthy_predicted	281	665

Table 2. Confusion matrix for the logistic regression on the features selected by the stepwise method

	Sick_true	Healthy_true
Sick_predicted	313	201
Healthy_predicted	289	662

Table 3. Confusion matrix for the logistic regression on the features selected by the MOGA

	Sick_true	Healthy_true
Sick_predicted	314	181
Healthy_predicted	288	682

Table 4. Confusion matrix for the SVM model on the full dataset:

a) SVM, the degree of a polynomial kernel = 1.0

	Sick_true	Healthy_true
Sick_predicted	342	242
Healthy_predicted	260	621

b) SVM, the degree of a polynomial kernel = 1.5

	Sick_true	Healthy_true
Sick_predicted	352	347
Healthy_predicted	250	516

c) SVM, the degree of a polynomial kernel = 2.0

	Sick_true	Healthy_true
Sick_predicted	360	340
Healthy_predicted	242	523

Table 5. Confusion matrix for the SVM model on the features selected by the MOGA:

a) SVM, the degree of a polynomial kernel = 1.0

	Sick_true	Healthy_true
Sick_predicted	306	173
Healthy_predicted	296	690

b) SVM, the degree of a polynomial kernel = 1.5

	Sick_true	Healthy_true
Sick_predicted	311	162
Healthy_predicted	291	701

c) SVM, the degree of a polynomial kernel = 2.0

	Sick_true	Healthy_true
Sick_predicted	303	187
Healthy_predicted	299	676
