# Supplementary material for: Evolutionary methods for variable selection in the epidemiological modeling of cardiovascular diseases
Source: BioData Min. 2018 Aug 14;11:18. doi: 10.1186/s13040-018-0180-x (PMC6092817; doi:10.1186/s13040-018-0180-x)
Supplement: Supplementary file 2 — Table S6. The list of selected variables with non-zero ranks based on stepwise selection. The file includes the list of variables and their MOGA and stepwise selection ranks with Pearson correlation values. (PDF 236 kb) [file 13040_2018_180_MOESM2_ESM.pdf]

Table 6. The list of selected variables with non-zero ranks based on stepwise selection

Variable description	Ranks		
	MOGA	Stepwise selection	Pearson correlation
Cigarettes/day *years of smoking	1.000	0.807	0.164
Carotid artery internal wall maximum thickness (mm)	0.999	0.766	0.206
Mean diastolic blood pressure (mmHg)	1.000	0.757	0.199
Age (years)	0.999	0.661	0.233
Diabetes (yes, no)	0.999	0.635	0.138
Maximal oxygen uptake (ml/kg/min)	1.000	0.615	-0.238
Hair mercury (μg/g)	0.997	0.573	0.149
Serum triglyceride concentration measurement year	1.000	0.445	-0.150
Serum linolenic acid (% fatty acids)	0.645	0.436	-0.067
LDL-cholesterol (mmol/l)	1.000	0.402	0.151
Plasma potassium (mmol/l)	0.296	0.385	0.055
Cheese consumption (g/day)	1.000	0.291	-0.169
Hypertension in family (yes, no)	0.306	0.290	-0.055
High-fat milk consumption (g/day)	0.997	0.280	0.130
Folate intake (μg/day)	1.000	0.265	-0.151
Plasma fibrinogen (g/l)	1.000	0.257	0.168
Polyunsaturated to saturated fatty acids intake ratio	0.106	0.256	-0.047
Sucrose intake (g/day)	0.906	0.252	-0.085
Plasma ascorbic acid (mg/l)	1.000	0.218	-0.184
Apple consumption (g/day)	0.006	0.216	0.022
Serum C-reactive protein (mg/l)	0.994	0.204	0.115
Wine intake (category)	0.085	0.200	-0.028
Mean systolic blood pressure (mmHg)	1.000	0.192	0.219
Serum testosterone, free (pmol/l)	0.005	0.191	0.025
Hypertension (yes, no)	1.000	0.184	0.192
Serum total cholesterol (mmol/l)	1.000	0.167	0.148
Body mass index (kg/m2)	1.000	0.158	0.165
Serum albumin (g/l)	0.987	0.158	-0.103
Plasma calcium, active, (mmol/l)	0.987	0.152	0.107
Serum oleic acid (% fatty acids)	0.490	0.152	-0.067
Serum fructosamine (μmol/l)	0.996	0.150	0.119
Serum triglycerides measurement time	0.007	0.148	-0.023
ECG P-Q interval (ms)	0.069	0.145	0.019
Serum total n-6 fatty acids intake (mg/day)	0.001	0.143	0.005
Pear consumption (g/day)	0.291	0.142	-0.054
Urine sodium (mmol/l)	0.151	0.129	0.046
Waist circumference (cm)	0.999	0.127	0.133
Beta blocker medication (yes, no)	1.000	0.121	0.121
ApoE phenotype 2-4 (yes, no)	0.003	0.121	-0.022
Fructose intake (g/day)	1.000	0.117	-0.130
High-fat cheese consumption (g/day)	0.999	0.115	-0.123
ApoAIV phenotype 11-21 (yes, no)	0.047	0.114	0.043
Cancer history (yes, no)	0.112	0.109	-0.042
Cranberry consumption (g/day)	0.087	0.103	0.015
Metabolic syndrome (yes, no)	1.000	0.100	0.141
Peonidin intake (mg/day)	0.009	0.100	0.012
Pyridoxine B6 intake (mg/day)	0.032	0.097	-0.040
Blood erythrocyte count (10**12/l)	0.002	0.093	0.006
High-fat unripened cheese consumption (g/day)	0.004	0.091	-0.009

Low-fat cheese consumption (g/day)	0.017	0.085	-0.033
Liquor drinker (yes, no)	0.003	0.083	0.000
ECG Q-T interval (ms)	0.004	0.075	0.008
Leisure time physical activity energy expenditure (kcal/d)	0.007	0.071	0.000
Jam consumption (g/day)	0.996	0.069	-0.117
Glucose intake (g/day)	0.972	0.067	-0.103
Shrimp consumption (g/day)	0.036	0.064	-0.036
Serum ApoA1 (g/l)	0.005	0.062	0.023
Serum insulin (mU/l)	0.999	0.058	0.144
Roots consumption (g/day)	0.926	0.057	-0.088
Cigarettes/day (n)	1.000	0.051	0.137
Marital status (category)	0.953	0.050	0.095
Canned fish consumption (g/day)	0.373	0.048	-0.059
Cysteine intake (mg/day)	0.000	0.042	-0.014
Serum uric acid (mmol/l)	0.958	0.041	0.098
Hypertension medication (yes, no)	0.999	0.036	0.134
Vitamin E supplement user (yes, no)	0.419	0.034	-0.055
Waist to hip ratio	0.998	0.033	0.153
Stroke in family (yes, no)	0.638	0.030	0.066
Selective betablocker medication	0.764	0.029	0.070
Secoisolariciresinol intake (µg/day)	0.002	0.028	0.004
Serum enterolactone (nmol/l)	0.009	0.025	0.016
Serum eicosapentaenoic acid (% fatty acids)	0.684	0.024	0.072
Lycopene intake (µg/day)	0.003	0.021	-0.007
Malvidin intake (mg/d)	0.009	0.018	0.016
Serum testosterone (nmol/l)	0.122	0.017	0.051
Tomato sauce (g/day)	0.008	0.014	0.003
Neurosis medication (yes, no)	0.056	0.012	-0.032
Methionine intake (mg/day)	0.024	0.009	0.028
Rapeseed oil consumption (g/day)	0.024	0.007	-0.040
Blood glutationperoxidase (mU/gHb)	0.003	0.007	0.004
Milk consumption (g/day)	0.784	0.006	0.079
