# Supplementary material for: Evolutionary methods for variable selection in the epidemiological modeling of cardiovascular diseases
Source: BioData Min. 2018 Aug 14;11:18. doi: 10.1186/s13040-018-0180-x (PMC6092817; doi:10.1186/s13040-018-0180-x)
Supplement: Supplementary file 3 — Table S7. The list of variables with MOGA-ranks ≥ 0.9. (PDF 245 kb) [file 13040_2018_180_MOESM3_ESM.pdf]

Table 7. The list of variables with MOGA-ranks ≥ 0.9

Variable description	Ranks		
	MOGA	Stepwise selection	Pearson correlation
Cigarettes/day *years of smoking	1.000	0.807	0.164
Mean diastolic blood pressure (mmHg)	1.000	0.757	0.199
Maximal oxygen uptake (ml/kg/min)	1.000	0.615	-0.238
Serum triglyceride concentration measurement year	1.000	0.445	-0.150
LDL-cholesterol (mmol/l)	1.000	0.402	0.151
Cheese consumption (g/day)	1.000	0.291	-0.169
Folate intake (µg/day)	1.000	0.265	-0.151
Plasma fibrinogen (g/l)	1.000	0.257	0.168
Plasma ascorbic acid (mg/l)	1.000	0.218	-0.184
Mean systolic blood pressure (mmHg)	1.000	0.192	0.219
Hypertension (yes, no)	1.000	0.184	0.192
Serum total cholesterol (mmol/l)	1.000	0.167	0.148
Body mass index (kg/m2)	1.000	0.158	0.165
Beta blocker medication (yes, no)	1.000	0.121	0.121
Fructose intake (g/day)	1.000	0.117	-0.130
Metabolic syndrome (yes, no)	1.000	0.100	0.141
Cigarettes/day (n)	1.000	0.051	0.137
Adulthood socioecomic status score (points)	1.000	0.000	0.179
Blood glucose (mmol/l)	1.000	0.000	0.147
Serum copper (mg/l)	1.000	0.000	0.154
Serum palmitoleic acid (% fatty acids)	1.000	0.000	0.126
Education (years)	1.000	0.000	-0.153
Income (Finnish markka)	1.000	0.000	-0.148
Age (years)	0.999	0.661	0.233
Serum apoliporotein B (g/l)	0.999	0.000	0.129
Serum insulin (mU/l)	0.999	0.058	0.144
Diabetes (yes, no)	0.999	0.635	0.138
High-fat cheese consumption (g/day)	0.999	0.115	-0.123
High-fat non-fermented dairy consumption (g/day)	0.999	0.000	0.121
Hypertension medication (yes, no)	0.999	0.036	0.134
Carotid artery internal wall maximum thickness (mm)	0.999	0.766	0.206
Carotid artery internal wall mean thickness (mm)	0.999	0.000	0.186
Smoker (yes, no)	0.999	0.000	0.118
Mean waist circumference (cm)	0.999	0.127	0.133
Urine nicotine metabolites (mg/day)	0.998	0.000	0.115
Waist to hip ratio	0.998	0.033	0.153
Blood leukocytes count (10**9/l)	0.997	0.000	0.117
Total carbohydrates intake (g/day)	0.997	0.000	-0.119
Glycemic load	0.997	0.000	-0.120
Hair mercury (µg/g)	0.997	0.573	0.149
High-fat milk consumption (g/day)	0.997	0.280	0.130
Caroten intake (µg/day)	0.996	0.000	-0.102
Serumfructosamine (µmol/l)	0.996	0.150	0.119
HDL/LDL cholesterol ratio	0.996	0.000	-0.110
Jam consumption (g/day)	0.996	0.069	-0.117
Total trans-polyunsaturated fatty acids intake (g/day)	0.996	0.000	0.108
Duration of hypertension (years)	0.995	0.000	0.100
Serum C-reactive protein (mg/l)	0.994	0.204	0.115
HDL2/LDL-cholesterol ratio	0.994	0.000	-0.099
Serum linolenic acid (% fatty acids)	0.993	0.000	-0.110
Forced vital capacity (l)	0.993	0.000	-0.134

Vegetables consumption, fresh and frozen (g/day)	0.992	0.000	-0.111
Serum albumin (g/l)	0.987	0.158	-0.103
Plasma calcium, active (mmol/l)	0.987	0.152	0.107
Saturated fatty acids intake (g/day)	0.987	0.000	0.095
Vitamin C intake (mg/day)	0.985	0.000	-0.102
Urine nicotine metabolites (mg/l)	0.980	0.000	0.096
Total fat intake (g/day)	0.979	0.000	0.093
Butter consumption (g/day)	0.978	0.000	0.094
Maltose intake (g/day)	0.978	0.000	0.089
Glucose intake (g/day)	0.972	0.067	-0.103
Fast respiratory volume at 1 second (l)	0.971	0.000	-0.110
Cruciferous vegetables consumption (g/day)	0.968	0.000	-0.092
Diuretic medication (yes, no)	0.964	0.000	0.091
Serum uric acid (mmol/l)	0.958	0.041	0.098
Marital status (category)	0.953	0.050	0.095
Non-sucrose carbohydrates intake (g/day)	0.949	0.000	-0.090
C18-3 linolenic acid intake (g/day)	0.944	0.000	-0.089
Salmon and lake fish consumption (g/day)	0.942	0.000	0.093
Diabetes medication (yes, no)	0.936	0.000	0.081
Alcohol consumption (dose/week)	0.931	0.000	0.088
Roots consumption (g/day)	0.926	0.057	-0.088
Alcohol binging (yes, no)	0.916	0.000	0.086
Water-soluble non-cellulose fiber intake (g/day)	0.914	0.000	-0.086
Sucrose intake (g/day)	0.906	0.252	-0.085
Vitamin D intake (µg/day)	0.902	0.000	0.091
